# Comparison of surgical outcomes between patients undergoing trans-oral endoscopic thyroid surgery combined with trans-thoracoareolar approach and conventional open surgery

**DOI:** 10.3389/fsurg.2022.916874

**Published:** 2022-09-08

**Authors:** Youyuan Shi, Lu Zhang, Chang Liu, Yong Wang, Hailin Zhang, Xiuan Lin

**Affiliations:** ^1^ Department of Head and Neck Surgery, Fujian Medical University Cancer Hospital, Fujian Cancer Hospital, Fuzhou, China; ^2^ Department of Head and Neck Surgery, Hunan Cancer Hospital, Changsha, China

**Keywords:** trans-oral endoscopic thyroid surgery, trans-thoracoareolar approach, conventional open surgery, comparison, outcomes

## Abstract

Over the past decades, remote-access thyroid surgery has been widely developed in the treatment of thyroid carcinoma, which can help patients to avoid cosmetically displeasing scarring. In this research, we collected and review our experience with endoscopic thyroidectomy with neck dissection *via* trans-thoracoareolar approach combined with trans-oral approach over a 3-year period. They were all diagnosed with thyroid carcinoma, and two of them had a complication of Hashimoto's thyroiditis. No patients were dissatisfied with the postoperative cosmetic results. One patient had numbness in the lower lip, but the symptom disappeared 1 month later. No infection, hemorrhage, or air embolism occurred. Pain and numbness in the endoscopic thyroid surgery group were slighter than in those who had open surgery. The present study aims to compare the feasibility and safety of trans-thoracoareolar approach combined with trans-oral approach to conventional open surgery.

## Introduction

Over the past decades, remote-access thyroid surgery has been widely developed in the treatment of thyroid carcinoma, which can help patients to avoid cosmetically displeasing scarring. Among them, the Trans-thoracoareolar approach has become the first choice by many surgeons, given the excellent cosmetic results with effective lesions oncologically resected when performing total endoscopic surgery for thyroid carcinoma (1, 2). For surgeons with proficient endoscopic operation techniques, under the premise of mastering the open combined radical resection of thyroid cancer, it is completely possible to perform endoscopic combined radical resection of thyroid cancer.

Due to the interference of the sternum stem and the clavicle, the thyroidectomy with neck dissection (TND) *via* trans-thoracoareolar approach cannot completely remove the grade IV and VI lymph nodes, which may lead to postoperative recurrence. For the similar reason, the lymph nodes in level II and upper level III cannot be removed completely under TND *via* trans-oral approach.

In this research, we collected and review our experience with endoscopic TND *via* trans-thoracoareolar approach combined with trans-oral approach over a 3-year period. The present study aims to compare the feasibility and safety of trans-thoracoareolar approach combined with trans-oral approach to conventional open surgery.

## Materials and methods

### Patients' criteria

Between June 2015 and December 2020, 40 female patients with ages ranging from 17 to 49 years were included in this study (Table 1).

**Table 1:** Clinical characteristics of including patients of this cohort.

Patient	Age	Tumor size (cm)	LN metastasis		Total operation time (mm)	Postoperative hospital (days)
			CC	LC		
1	17	2.5	2/7	4/18	360	6
2	22	1.5	3/8	5/25	229	5
3	33	2.5	1/7	3/19	260	5
4	21	2.0	2/8	0/22	300	5
5	35	1.5	3/9	6/21	320	6
6	29	2.5	4/7	7/28	330	6
7	33	1.0	1/3	2/18	290	5
8	35	2.0	2/6	3/19	310	5
9	26	1.9	3/7	5/28	330	6
10	22	1.5	2/6	4/19	309	5
11	41	1.0	1/4	3/18	300	4
12	38	1.8	4/7	4/22	330	5
13	49	1.6	2/5	2/18	280	4
14	32	1.6	2/7	4/19	340	5
15	27	1.5	4/8	6/29	360	6

Inclusion criteria were as follows: single or multiple tumors located in the gland without invasion of the capsule, diameter <3.0 cm; patients diagnosed with thyroid carcinoma by ultrasonic, CT, or fine needle aspiration preoperatively; and lateral neck lymph node metastasis revealed by imaging or fine needle aspiration without confluent tumors or invasion of the internal jugular vein. (1) Endoscopic surgery was performed on 20 patients *via* trans-thoracoareolar approach combined with trans-oral approach. Seventeen of them were diagnosed with a single tumor, nine in the left and eight in the right. Three of them were diagnosed with multiple tumors. The maximum diameter did not surpass 2.5 cm. The largest lymph node diameter is 1.5 cm without fusion or invasion of the internal jugular vein. (2) For the conventional open groups, 16 of them were diagnosed with a single tumor, 10 in the right and six in the left. Four of them were diagnosed with multiple tumors. The condition of the lymph nodes is similar to that of the endoscopy group.

Exclusion criteria were as follows: tumor located in the upper pole of the posterior aspect of thyroid, close to recurrent laryngeal nerve; invasion of the capsule and surrounding tissues such as recurrent laryngeal nerve, trachea, esophageal, or belt-shaped muscle; lymph node metastasis with confluent tumors or invasion of the internal jugular vein; and patients a history of thoracic or neck surgery.

We defined transient hypocalcemia and transient recurrent laryngeal nerve injury based on the recovery from symptoms and normalization of laboratory data within 6 months.

All the patients had taken levothyroxine for thyroid stimulating hormone suppression after the operation and were regularly followed up with serum thyroglobulin (Tg) and neck ultrasonography at intervals of 3 months or 6 months.

The mean follow-up period was 26.4 months (range, 9–42 months). No evidence of residual or recurrent disease was found at follow-up. The cosmetic results of this procedure were excellent.

### Operative techniques

The transnasal endotracheal intubation was adopted for general anesthesia. Antibiotics were used once 30 min before operation. Patients were in a supine position with head hypsokinesis, and their necks were straightened with cushioning being placed under the shoulder so that the necks were in a slight hyperextension position.

The working space was established *via* trans-thoracoareolar approach. The operator stood between the thighs of patients. The “inflation fluid” was prepared with adrenaline and saline (the dilution ratio is 1:500). Two incisions were made at 11 o'clock and 4 o'clock of the left areola, respectively, the length of the former is 0.5 cm, and the length of the later is 1 cm. Another 0.5 cm long incision was made at 11 o'clock of the right areola, dissection would not be ceased until the deep fascia was exposed. The “inflation fluid” was subcutaneously injected into the neck area where the flap was separated from *via* the incision previously made, about 100–200 mL of “inflation fluid” was injected with a special needle connected with a 50 mL syringe. Blunt dissection between superficial and deep fascia was accomplished with visual Trocar, the operation tunnel or space would not be established until reaching the clavicles. Excess liquid was squeezed out later. A 10 mm Trocar was inserted *via* the incision at 4 o'clock of the left areola, and then a 30° laparoscope was inserted through the Trocar. CO_2_ was insufflated with the pressure maintained at about 6–8 mmHg. After that, two 5 mm Trocars were inserted *via* the incision at 11 o'clock of the left and right areolas, respectively, an operating forceps and an ultrasonic scalpel were inserted through the two Trocars accordingly.

**Figure 1:**
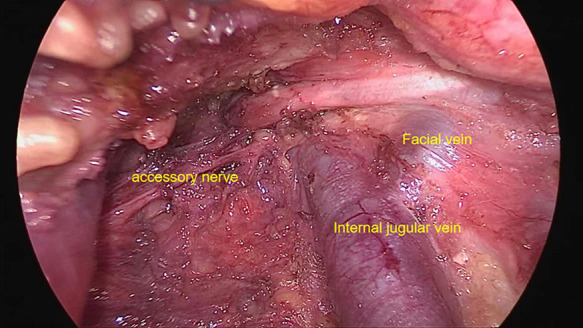
Area II lymph node dissection *via* trans-thoracoareolar approach.

Under the monitoring of a laparoscope, dissection was made between subcutaneous tissue and deeper tissue in the chest and neck. When reaching the neck area, dissociation was made between the platysma and deep loose connective tissue. Dissociation would not be stopped until reaching the hyoid and the lateral margin of sternocleidomastoids. Using an ultrasonic scalpel, the incision was made in linea alba cervicalis and the surgical capsule of thyroid sequentially. Dissociation was made between the two layers of capsule of thyroid, and then the thyroid was exposed. To perform unilateral lobectomy or to perform bilateral thyroidectomy, the decision was made according to preoperative ultrasonic, CT results, and operation findings. After that, dissection was continued. When reaching the inferior margin of submandibular glands, dissection was made 2 cm outwards of the lateral margin of sternocleidomastoids. Using an ultrasonic scalpel, the incision was made between the sternal head and clavicular head of the sternocleidomastoid, and then traction was made with an endoscopic retractor. Omohyoid was cut off subsequently. When the vascular sheath was cut with a scissor and exposed, dissociation was made among arteria carotis communis, vena jugularis interna, and nervi vagus. The lateral neck lymph nodes were removed from the bottom up along vena jugularis interna, removing would not be ceased until the lymph nodes in level II were completely removed (Figure 1). The C2, C3, C4, and accessory nerves were preserved, meanwhile, the thoracic duct and right lymphatic duct were protected.

**Figure 2:**
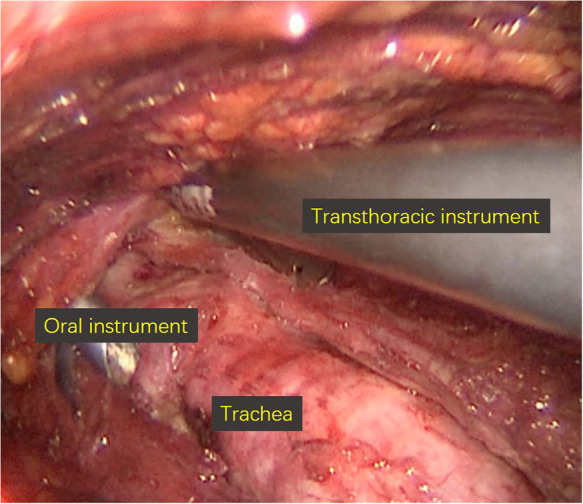
Working space *via* trans-oral approach.

**Figure 3:**
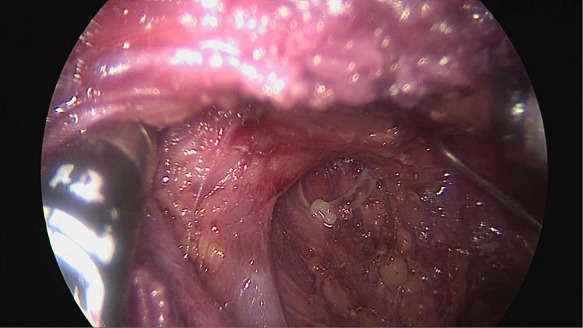
Area II lymph node dissection *via* trans-oral approach.

The other working space was established *via* trans-oral approach (Figure 2). A 1 cm long incision was made at the midpoint of sulcus vestibularis, and two 0.5 cm long incisions were made in front of the left and right first premolars in sulcus vestibularis. Trocars were inserted *via* the three incisions previously made. Since the working space in the neck was established before, it is easy to enter the field. The lymph nodes in level VI and level VII were removed, and the residual lymph nodes in level IV which were neglected during the removal *via* trans-thoracoareolar approach were eventually cleared away (Figure 3). At the end of the operation, the specimen was put into specimen bags and extracted, the operation cavity was washed with distilled water, and drainage tubes were placed.

### Tips and key points

For male patients, the chest incision is suggested to be made in the second intercostal space, if so, the wound will be minimized, and the operation will be facilitated. For female patients, if the chest incision was hidden into the aureola, the scars will be invisible postoperatively. The lymph nodes in level IIB should be removed conventionally, and the removal should not be ceased until the surface of splenius capitis is exposed.

As for the scope of operation, the flap needs to be separated from the inferior margin of glandula submandibularis upwards, to the superior margin of clavicle downwards, to the medial margin of the contralateral sternocleidomastoid inwards, and the posterior margin of sternocleidomastoid outwards. When reaching the posterior margin of sternocleidomastoid, dissection should be made 2 cm outwards again. Some authors proposed that the marginal mandibular branches of the facial nerve may descend 2 cm inferior to the inferior margin of glandula submandibularis if the skin in the patient's neck is very loose (8). But in fact, the marginal mandibular branches of the facial nerve descend for the loose skin, it distributes in the deep surface of platysma and locates in the region where it is 1.2 cm superior or 0.8 cm inferior to the inferior margin of mandibular. We had never seen the marginal mandibular branches of the facial nerve descend further than 2 cm intraoperatively, so the separation mentioned above would not be threatening (Table 2).

**Table 2:** Endoscopic lateral neck dissection reported by other authors.

Author	Number of cases	Technique	Type of operation	Mean lymph nodes yield	Means operative time (min)	Complications	Means PTH (days)
Li et al. (9)	11	SET	SLND	26	94	None	4.3
Miccoli et al. (10)	2	VAT	SLND	8.5	NA	None	NA
Kitagawa et al. (11)	3	VAT	MRND	NA	264	None	6.5
Lombardi et al. (12)	2	VAT	FLND	25	154	TH	5
Kang et al. (13)	13	AP	MRND SLND	18.8	286	NA	5.3

VAT, video-assisted thyroidectomy; SLND, selective lateral neck dissection; MRND, modified radical neck dissection; FLND, functional lateral neck dissection; TH, transient hypoparathyroidism; NA, not available.

When the separation of the flap is accomplished, the dissection of sternocleidomastoid should be started sequentially. The traction of the anterior margin of sternocleidomastoid should be made outwards with an endoscopic retractor, and then dissection should be continued outwards. For the restriction of retractor, the lower part of sternocleidomastoid cannot be dissected sufficiently, you can only touch the vena jugularis interna. The space sternal head and clavicular head of sternocleidomastoid should be expanded, and these heads should be retracted laterally with retractors. The sternocleidomastoid should be completely dissociated. Omohoid should be dissociated, but not cut, because it can not only provide traction for other tissue in its affinity but also help the surgeon to determine the scope of operation as a landmark. The neck dissection should be performed from upside to downside, and from the middle to the two sides.

The accessory nerve should be correctly identified. First, dissociation should be made along the lateral margin of the posterior belly of digastrics, when reaching the lateral side of vena jugularis interna, the outlet where accessory nerve exits from the skull will be exposed. Second, Dissociation should be made along the sternocleidomastoid, when reaching the lateral side of vena jugularis interna, the intlet where the accessory nerve enters into the sternocleidomastoid will be exposed. Dissection should be continued upwards and the accessory nerve should be completely exposed. Third, if it is needed to perform neck dissection in level V, when the great auricular nerve is dissociated and dissection is made 1 cm upwards, the intlet where the accessory nerve enters into the sternocleidomastoid will be exposed.

The rupture of the internal jugular vein and thoracic duct should be correctly handled. First, we encountered one case of rupture of the internal jugular vein in this study. Do not attempt to make occlusion with the vessel clamp, or the rupture will be expanded. Suction should be made sufficiently, thus the surgical field can be clearly observed. The “8” pattern of stitching should be accomplished with 4-0 suture one-handed, and then suction should be ceased. Knot tying should be accomplished using both hands under a laparoscope. Otherwise, when you encounter the rupture of the thoracic duct, dissociation should be made carefully and occlusion should be made with a vessel clamp *via* trans-oral approach. Occlusion should not be made *via* trans-thoracoareolar approach, for there is an obstruction caused by the clavicle. It is safe and convenient to operate *via* trans-oral approach.

### Postoperative management

After surgery, antibiotics were applied for 2 days, and benzalkonium bromide mouthwash was given to rinse the mouth after each meal. Liquid diet was allowed on the first day after surgery, and then semi-liquid diet was allowed on the second day after surgery. There is no need to deal with pain or numbness in the incisions, these symptoms will disappear in 1 month usually. Normally, swelling is not severe, so no measures should be taken to deal with it.

## Discussion

With the development of endoscopic thyroid surgery technology and the patients' desire for cosmetic results, the indications of endoscopic thyroid surgery have expanded from benign thyroid nodule to thyroid carcinoma with lymph node metastasis. Although there is a lack of large-sample randomized controlled studies, recent follow-ups showed that no difference was found in thyroglobulin level and recurrence rate between patients with endoscopic surgery and open surgery (3–6). Therefore, the technique of endoscopic surgery should be applied not only in thyroid carcinoma with lymph node metastasis in the central cervical area but also in lymph node metastasis in the lateral cervical area, in which neck dissection under endoscope leads to authentic cosmetic and minimally invasive results. In the past years, endoscopic thyroid surgery was thought to be extremely invasive for the large scope of operation. However, patients who underwent neck dissection in the lateral cervical area with long L-shaped incisions suffered tremendous physiological and mental pain. On this account, neck dissection in the lateral cervical area under endoscope is an ideal method, because it is mentally minimally invasive. Cases of total endoscopic neck dissection in the lateral cervical area have been reported by Wang et al. (7), which demonstrates that endoscopic surgery not only leads to better cosmetic results but also clearer re-movement in level IIB because of the magnification effect of the endoscope. What's more, due to the small scope of separation brought by the endoscope, the trauma is less than that of open surgery.

Recently, some authors performed endoscopic neck dissection in the lateral cervical area *via* trans-thoracoareolar approach. However, due to the existence of the anatomical blind area, especially the affinity of the clavicle and level II, it is hard to remove the affected lymph nodes, which may lead to unmanageable hemorrhage or lymphatic fistulas and residue of lymph nodes. These problems will be solved with endoscopic operation *via* trans-oral approach. As for the total trans-oral approach, the lymph nodes removed were only limited to level VI. In addition, it is difficult to learn to operate by this method, which will cost a long learning curve. But if the working space is established *via* trans-thoracoareolar approach in advance, it may be easier to operate *via* trans-oral approach. Moreover, it is very difficult to remove the lymph nodes in levels II, III, and IV *via* trans-oral approach. But it is easier to operate *via* trans-oral approach if the dissociation is made sufficiently *via* trans-thoracoareolar approach in advance. In our study, we found that if the dissociation of the posterior margin of the sternocleidomastoid achieves 2 cm, the lymph nodes in level V is not difficult to remove. The combination of these two approaches has advantages in removing the lymph nodes in the lateral neck area, which will benefit surgeons with little experience in oral operations. Thus, the difficulty of learning for endoscopic thyroid operation is greatly reduced.

In our study, when we were removing the lymph nodes in level IV *via* trans-oral approach, residual lymph nodes left by operation *via* trans-thoracoareolar approach were discovered in five patients, and lymph node metastasis was found in three of these five. The other four patients were also diagnosed with lymph node metastasis in the progress of removing the lymph nodes in level IV *via* trans-oral approach. Thus, there is a possibility of residue in removing lymph nodes *via* total trans-thoracoareolar approach. Rupture of thoracic duct was found in one patient when we were removing the lymph nodes *via* trans-oral approach. Fortunately, we made occlusion with vessel clamp *via* trans-oral approach later, and no lymphatic fistulas occurred after surgery (Figure 3). Both the total trans-thoracoareolar approach and total trans-oral approach have their own disadvantages. The total trans-thoracoareolar approach is not always efficient with complete dissection of the lymph nodes, which is affected by the skill of surgeons and the tumor-nodemetastasis stage. However, the combination of trans-thoracoareolar approach and trans-oral approach could achieve this goal easily. Moreover, compared with open surgery, the combination of these two approaches not only achieves significant cosmetic effects but also causes less tissue damage and has shorter hospital stays. It is important to be clear that not every patient is amenable to the endoscopic approach, given that the radical cure of cancer is always in the first place. Thus, we should carefully control indications and contraindications for the endoscopic thyroidectomy. Hence we propose to combine these two approaches to improve the endoscopic surgery and curative effect.

In conclusion, to have endoscopic surgery for thyroid carcinoma *via* the combined approach, the candidate should meet the requirements below: (1) Age <45 years old. If a patient is eager for cosmetic results, this requirement can be relaxed. (2) The diameter of thyroid mass should not be larger than 3 cm. No affection in the adjacent tissue. (3) No widespread lymph node metastasis, confluent tumors, or invasion of the great vessels in the neck. (4) Eagerness for cosmetic results.

## Conclusion

All of the patients had undergone successful surgery. They were all diagnosed with thyroid carcinoma, and two of them had a complication of Hashimoto's thyroiditis. No patients were dissatisfied with the postoperative cosmetic results. One patient had numbness in the lower lip, but the symptom disappeared 1 month later. No infection, hemorrhage, or air embolism occurred. Pain and numbness in the endoscopic thyroid surgery group were slighter than in those who had open surgery.

## Data Availability

The original contributions presented in the study are included in the article/Supplementary Material, further inquiries can be directed to the corresponding authors.
